# Development of a theoretical framework for analyzing cerebrospinal fluid dynamics

**DOI:** 10.1186/1743-8454-6-12

**Published:** 2009-09-22

**Authors:** Benjamin Cohen, Abram Voorhees, Søren Vedel, Timothy Wei

**Affiliations:** 1 Mechanical, Aerospace and Nuclear Engineering, Rensselaer Polytechnic Institute, 110 8th Street, Troy, NY 12180, USA; 2 Siemens Healthcare, 51 Valley Stream Parkway, Malvern, PA 19355, USA; 3 Department of Micro- and Nanotechnology, Technical University of Denmark, DTU Nanotech Building, 345 East, DK-2800 Kongens Lyngby, Denmark

## Abstract

**Background:**

To date hydrocephalus researchers acknowledge the need for rigorous but utilitarian fluid mechanics understanding and methodologies in studying normal and hydrocephalic intracranial dynamics. Pressure volume models and electric circuit analogs introduced pressure into volume conservation; but control volume analysis enforces independent conditions on pressure and volume. Previously, utilization of clinical measurements has been limited to understanding of the relative amplitude and timing of flow, volume and pressure waveforms; qualitative approaches without a clear framework for meaningful quantitative comparison.

**Methods:**

Control volume analysis is presented to introduce the reader to the theoretical background of this foundational fluid mechanics technique for application to general control volumes. This approach is able to directly incorporate the diverse measurements obtained by clinicians to better elucidate intracranial dynamics and progression to disorder.

**Results:**

Several examples of meaningful intracranial control volumes and the particular measurement sets needed for the analysis are discussed.

**Conclusion:**

Control volume analysis provides a framework to guide the type and location of measurements and also a way to interpret the resulting data within a fundamental fluid physics analysis.

## Background

The central nervous system consists of the brain and spinal cord which are bathed in a clear, nearly cell-free fluid termed cerebrospinal fluid (CSF). CSF resides in the subarachnoid spaces surrounding the brain and spinal cord, and in four fluid reservoirs within the brain known as the cerebral ventricles. The primary mechanical role of CSF is protecting the brain from abrupt movements and transmitting arterial volume displacement to venous blood which attenuates the intracranial pressure (ICP) pulse. Intracranial dynamics is defined as the complex physical coupling between the brain and the cerebral blood and CSF flows and pressures. A departure from equilibrium intracranial dynamics may lead to hydrocephalus, a complex spectrum of diseases, primarily defined by perturbation of the cranial contents (CSF volume) and ICP.

The original theories of hydrocephalus, as a derangement of CSF bulk flow, an imbalance in production (by the choroid plexus) and absorption (or outflow resistance), led to the invention and ultimate adoption of the shunt as the primary treatment option. Indeed individuals experienced improvements after shunting, but shunts were not without complication (in fact have introduced new ones) and, as pointed out by Madsen *et al*., may have slowed progress, as hydrocephalus was viewed as a solved problem [1]. Over the past two decades the bulk flow interpretation has been scrutinized as improvements in flow-sensitive magnetic resonance imaging (MRI) have shown pulsatile CSF flow at nearly all intracranial sites [2]. While much of the hydrocephalus research community remains divided as to the relative importance of the bulk and pulsatile components of CSF flow there is increasing interest across this divide to obtain and interpret clinical data to improve physiological understanding and mathematical modeling of disease development.

### Past models of the intracranial space

Mathematical models of the intracranial space are important to our understanding of hydrocephalus. Historically modeling has been accomplished by simplifying assumptions as to the physical mechanisms of primary importance. In the case of hydrocephalus, examples of these assumptions would be: 1) CSF is an incompressible, Newtonian fluid, 2) most is produced by the choroid plexus at a constant rate, 3) CSF absorption occurs by a "valve" mechanism into the capillaries (in cases, specifically through the arachnoid granulations) 4) compliance represents the volume storage capacity of tissue as the CSF pressure changes, 5) the intracranial space has a fixed volume (i.e. the Monroe-Kellie doctrine), 6) CSF flow is governed by similar equations for current in an electric circuit. Many of these assumptions are based on previous experience and experiments, and are founded to varying degrees.

Numerous investigators have studied hydrocephalus using simplified mathematical models of the CSF spaces. Sivaloganathan *et al*. showed the commonality between all pressure volume models, derived a pressure evolution equation and provides various solutions based on the functional form of brain compliance [3]. CSF was modeled as an incompressible fluid and the single compartment's volume change was dictated by the mismatch in formation and absorption rate. An exponential pressure volume relationship, based on studies by Marmarou *et al*. [4,5], has received the most attention as a model of the intracranial compliance to date.

More detailed models compartmentalized the CSF system into a hydraulic network of pressure volume models, which were commonly understood through electrical circuit analogs [2,5-9]. Flow and pressure drop were related through the (hydraulic) resistance, in the same form found between current and voltage in an electric circuit (i.e. Ohm's Law). In this framework, large fluid spaces (e.g. ventricles or subarachnoid spaces) were modeled as lumped parameter compartments (pressure volume models) and narrow conduits between them as resistors (or impedance). Early circuit models of this type (i.e. [5,6]) studied bulk flow. More recent models, based on pulsatile flow, utilized the concept of complex impedance (phasors) from RLC electric circuit analysis to study the amplitude modulation and phase lag observed between arterial and CSF flow and pressure variations [2,10].

Hakim *et al*. [11] stressed the importance of understanding the intracranial cavity in terms of classical physics and mechanics concepts, prior to trying to fully explicate complex physiological mechanisms in terms of biological phenomenon. Mechanically, the brain parenchyma was viewed as a sponge of visco-elactic material with 'cells' of various sizes (e.g. intraparenchymal venous blood and extracellular spaces). A hollow spherical model of homogeneous elastic brain was used to theoretically determine the stress distribution to relate ventricular enlargement and transmantel pressure differences.

Advances in numerical methods and computational power led to still more detailed models of CSF flow and brain remolding; continuum based modeling. Nagashima *et al*. used finite elements to model the brain as a porous material with viscous fluid through flow to study the interstitial pressure, intracerebral stress distribution and the ventricular shape in a 2D axial slice [12]. Jacobson *et al*. [13] and Loth *et al*. [14] used the Navier-Stokes equations to study CSF flow in the aqueduct and spinal canal, respectively. Linninger *et al*. [15] solved the incompressible continuity and Navier-Stokes equations within segmented 2D sagittal slices from normal human subjects and patients. Darcy's Law and an inertial term amended the Navier-Stokes equation for CSF flowing within brain tissue, modeled as a porous media with volumetric CSF production. Absorption was assumed to occur solely through the arachnoid granulations, in disagreement with the view of Greitz that the brain capillaries absorb CSF diffusely [16]. Velocity and pressure along the domain boundaries were specified to compute CSF velocity and pressure at all points in the domain of study. With the full solution, the measured CSF flow at several locations was compared to the computational results, as model validation. Boundary conditions were chosen based on physiological arguments regarding the intracranial behavior and clinical data. The assumed conditions on the domain boundary and form of the governing equations greatly impact the solution; and therefore must be chosen carefully to obtain a realistic picture of the system being studied.

Investigations which seek to directly interpret clinical data (e.g. MRI, ICP monitoring, etc.) have been limited to presentations of relative amplitude and timing of flow, volume and pressure waveforms [10,17-22] or comprehensive descriptions of the intracranial dynamics [23,24] without a clear method of accounting or interpretation based on physics principles. Alperin *et al*. [25] used principles of volume conservation, results of baboon and computational studies [14], and assumed an exponential ICP-volume relation to noninvasively estimate ICP and intracranial elastance.

### Discrepancy between past models and control volume analysis

Regardless of the assumptions used to model the intracranial space, a clinician's decisions for diagnosis and treatment are based on, and limited by, the availability and interpretation of patient specific data in relation to the populations of healthy and diseased. Much has been learned about normal and hydrocephalic intracranial dynamics from previous investigations. Even so, advancements incorporating measurements within a precise, physically meaningful, direct physics analysis will improve understanding and quantification of disease progression and diagnosis. To date investigators acknowledge the need for elementary fluid mechanics in studying normal and hydrocephalic flow and pressure dynamics and the lack of a comprehensive and accurate quantification of the three-dimensional flow based on basic fluid physics [15].

Two assumptions of the pressure volume models, the absorption mechanism and brain compliance, changed the variable of interest from volume to pressure. This was advantageous because pressure was more readily available than volume information, however the assumed compliance affects the intracranial response and hence is an integral part of the phenomenological model. Likewise, circuit analogs introduced pressure into volume (mass) conservation principles; the pressure drop between fluid spaces and the connecting conduit's resistance (or more generally, impedance) dictates the volume flow rate. Therefore, flow through conduits was governed by the pressures within the adjacent compartments, which transiently depend on their compliance.

From a first principles fluids perspective mass and momentum conservation lead to independent constraints on the flow of fluid (in this case the primary concern is CSF and blood flow). Mass conservation, or for an incompressible substance volume conservation, determines flow rates while momentum conservation is used to determine the pressure in the flowing fluid. Therefore, utilizing compliance and hydraulic resistance to underscore complex intracranial processes to determine CSF pressure, flow and volume, while of clinical utility, my be an unwarranted simplification from a mechanics perspective. In control volume analysis these quantities are determined through separate equations. Continuum based models represent a step in the right direction, however control volumes can be used to account for the same physics in a more direct and simplified framework.

In order to improve our understanding of the normal intracranial state and progression to disorder, we seek to implement the most basic fluid dynamic analysis directly to control volumes within the cranium, allowing a direct mechanical interpretation of the data. The proposed framework is an elementary, foundational fluid mechanics formulation called integral control volume analysis [26,27]. The approach entails defining regions in the brain; in the fluid dynamics lexicon these are referred to as 'control volumes'. From a traditional fluid mechanics perspective it is a first analysis approach because it does not require a detailed knowledge of the entire flow. Conservation equations are written for each control volume being studied. Measurements based directly on the physical meanings of terms in the conservation equations dictate the type and location of data obtained and relates these to other measurements in the same individual. The requisite measurements are those of velocity and pressure at specific locations on the control volumes' surfaces and volume (change) measurements of the control volumes themselves. In effect, this is a budgeting procedure, using fundamental conservation laws (mass and momentum) to keep track of and account for important parameters in the cranium.

This paper introduces control volume analysis to the clinical community and explains how it may be used to better direct and interpret clinical examinations. While this approach has not been used in this context, past experience using control volume analysis prove it is a valid technique and theoretically limited only by the availability of clinical data. Future work involves proving the proposed framework may be used to deduce important parameters regarding intracranial dynamics by working with members of the community to decide upon the set of control volumes to which the budgets will be applied and developing the appropriate data sets. What follows is a description of the control volume framework, the measurement techniques employed, to date, to obtain the data sets needed for the control volume budgets and discussion of several plausible intracranial control volumes.

## Methods

Integral control volume analysis has been widely used among engineers and physicists to extract important physical information about structures and devices within fluid flow. In mechanical and aerospace engineering, control volumes are typically used to calculate the thrust produced by a jet engine based on the flow field through the engine. Likewise, lift on an airfoil (or drag generated by a obstruction to flow) can be computed based on knowledge of the velocity field. The same approach may be used to determine the acceleration of a rocket, rate of oil gain/loss from a hydraulic accumulator and the pressure drop across a sudden expansion in a pipe. These important parameters can be obtained with a minimal knowledge of the flow, hence the utility of the control volume formulation. When more detailed analysis is desired, such as the velocity and pressure distribution within the jet engine or on the surface of an airfoil, respectively, then the differential (continuum) forms of the conservation equations must be solved. It is important to note that in the limit as we create an infinite number of infinitesimally small control volumes, we will obtain the differential forms of the mass and momentum conservation equations, the Navier-Stokes equations (e.g. see Ref. [26]), commonly used in hydrocephalus research. Control volume analysis requires similar clinical inputs as continuum models, i.e. velocity, volume and pressure data, but determines overall behavior of the device without detailed knowledge of the entire flow.

### Theoretical development using fluid dynamics

Before proceeding with a discussion of control volume analysis applied to intracranial control volumes, it would be helpful to review the general theoretical underpinnings of the proposed fluid mechanics framework. The primary advantages of using control volume analysis (i.e. fixed region) as opposed to the system formulation (i.e. fixed mass) to study fluid flow are that: 1) the control volume and control surface are defined at all times and 2) the effect of flow on adjacent structures or devices may be determined and provides more pertinent information than the motion of a specified mass of fluid.

To start, a *system *may be defined as a collection of fluid particles comprising part of a flow of interest (e.g. a blob of ink, the system, in a hose, the flow of interest). The system boundaries (to continue the example, the outside surface of the ink blob) are such that the same fluid elements are always contained therein. Thus, even though the shape of the system will change in the flow as time progresses, the same particles will always be on the surface of the ink blob, separating ink inside from water outside. Necessarily, the mass of a system is constant, and it is in principle possible to write equations of motion for the system, i.e., solve for the system's motion given the applied forces.

**Figure 1:**
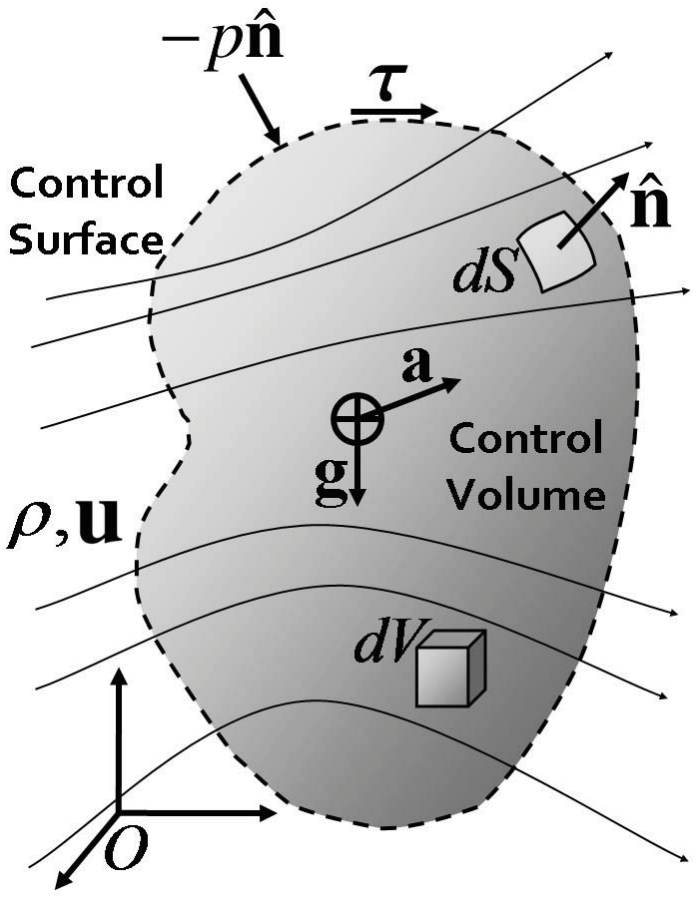
**Universal control volume for definition of terms**. A universal control volume represented by a shaded region within fluid of density *ρ *and velocity vector field **u**. The control surface is shown as dashed line at the boundary of the control volume. Differential volume and surface area elements, *dV *and *dS*, respectively, are shown along with corresponding unit outward surface normal vector . Body forces caused by gravity **g **and acceleration **a **act on the center of mass of the control volume, while pressure -*p* and viscous shear stresses ***τ ***act on the control surface, normal and tangentially, respectively. Reference frame *O *is a fixed inertial frame with respect to the control volume.

For many flows, however, such a formulation would be intractable or inconvenient at best, due to the difficulty of 'following' the evolving system (ink blob) as it flows (down the hose) and becomes increasingly distorted. For this reason, we define a *control volume *as a clearly defined space through which fluid may pass. Control volumes are mathematical regions used for analysis and typically coincide with some physical boundaries of the flow, but could be arbitrarily defined at the discretion of the user. Figure 1 shows a general or universal control volume within a fluid of mass density *ρ *with velocity field **u **flowing through. The external boundary of the control volume is referred to as the *control surface*, shown as a dotted boundary in the figure. The advantage of this approach is that the boundary of the control volume is prescribed at all times. Typically, one dictates that the control surfaces coincide with some physically meaningful boundary in the flow, e.g., the walls of the hose, so that no fluid will pass through them. In places where the control surface does not coincide with physical boundaries, such as at the inlet and outlet to the hose, fluid may flow through the control surface, which does not physically exist. For the flow of blood and CSF, it may be expedient to allow the control volume to move or change shape depending on the flow of interest (i.e. because the ventricle walls move during the cardiac cycle).

It is possible to write the rate of change of any property of a system in terms of control volume parameters. This is the classic Reynolds transport theorem [26,28], which states that the change of any extensive property (e.g. mass, momentum and energy) of the system equals the change of that property in the control volume and the amount flowing through the control surface at any given instant.

One can think of a control volume as a corral built to subdivide a rancher's pasture. In this analogy, the cranium would be the pasture. Depending on the rancher's needs, he/she will build as many or few corrals within the pasture as needed. So too with this research approach, we can define as many or as few control volumes as a function of the scientific drivers. Minimally, at least one control volume for the CSF and one for the blood is required to study the important interactions that define the intracranial state. Just as the rancher keeps track of animals entering and leaving through the corral gates and the total number of animals inside the corral, control volume analysis is simplified by the fact that only effects occurring at the control surface and net changes within the volume are required. The rancher need not account for every animal at all times, as the system formulation would require.

In order to maintain continuity for the reader the explanation of the integral mass and momentum conservation equations, as applied to a general control volume, will be presented in the following two subsections. The first is a physical explanation in words of the conservation equations. The latter, which may be skipped by readers not interested in the full mathematical description, is included for completeness. Both sections discuss the same material, from a written and mathematical prospective, respectively to fully explicate conservation principles as applied to control volumes.

#### Physical explanation of the integral conservation equations

Conservation laws are commonly used in physics to relate important variables of interest. Hydrocephalus research and modeling has been no exception. Pressure volume models [3] and electric circuit analogs [2,6] both enforce the condition that mass may not be created or destroyed, or mass conservation. However, using a change in variables, the mass (volume) constraint is written in terms of pressure. From basic fluid mechanics theory an additional constraint on the forces (and hence pressures) is required, as in Ref. [15], where mass and momentum conservation equations were solved numerically.

Mass conservation is simply the accounting of flow in and out of a control volume and the total mass within the control volume. Just like the rancher keeps track of animals entering and leaving through the corral gates and the total number of animals inside the corral, information regarding the fluid velocity through the control surface and the total amount of fluid inside the control volume is needed. Note, the total fluid mass (in a control volume) could change because of production/absorption (of CSF), or from changes in volume (e.g. pulsatile volume change of a ventricle or blood vessel). The conservation of mass equation for a control volume states that the time-rate of change of fluid mass within the control volume must equal the net mass flow into the control volume through the control surface plus the net production (production minus absorption) of mass within the control volume. This simplifies analysis because only flow velocities at the control surface and net changes within the control volume enter in the formulation. 

Newton's 2^*nd *^law stipulates conservation of momentum for all objects, including fluids. For a solid object of mass *m *exposed to the resultant force **F **it reads **F **= *m*(*d***u**/*dt*) where *d***u**/*dt *is the time-rate of change of the velocity, i.e. acceleration of the object. The resultant force **F **is the sum of all external forces acting on the object. This basic physical picture also applies to fluids, although the mathematics become more complicated due to the fact that fluids can flow. For a control volume, the time-rate of change of the momentum inside the control volume must equal the forces acting on the control volume and the forces created by fluid flow crossing the control surface. Forces act on a control volume when fluid flows through control surfaces, as can be experienced with a garden hose: defining the control volume as the total internal volume of the hose, you will find that the hose experiences a force when water exits it (i.e. one has to physically restrain the end of the hose from whipping about). The remainder of the forces on the control volume may be broken into surface and body forces. Surface forces, such as pressure and viscous shear (fluid friction) stresses, act on the control surface. Body forces, such as gravity and acceleration, act on the mass within the control volume. Contribution to these forces are shown in Figure 1 acting on the surface and center of gravity of the control volume, respectively. Dynamic postural changes are inherently included in the momentum equation; as individuals reposition their heads (e.g. sit up or lay down), both the acceleration and relative alignment with gravity will change. From experience, this will affect the flow and pressures in the control volume, which may be accounted for through the conservation equations. Pressure also drives fluid flow and therefore appears in the conservation of momentum equation, but only enters the analysis though its effect at the control surface.

#### Mathematical explanation of the integral conservation equations

The conservation of mass equation for a general control volume is written in mathematical form as:

(1)

where:

(*i*) Rate of change of fluid mass in the control volume

(*ii*) Net mass flow rate across the control surface

(*iii*) Net mass production/absorption rate within the control volume

Likewise, the conservation of momentum equations for a general control volume are written:

(2)

(3)

(4)

where:

(*i*) Rate of change of fluid momentum in the control volume

(*ii*) Net fluid momentum flow rate across the control surface; force due to fluid flow

(*iii*) Net body forces acting on mass in the control volume

(*iv*) Net surface forces acting on the control surface

Here we focus on the dominant terms; body forces (3) caused by gravity and acceleration and surface forces (4) due to pressure and viscous shear stresses, respectively.

In these equations the symbols represent physical quantities presented in Table 1 and displayed schematically in Figure 1. The equations and schematic follow the presentation in Ref. [26].

**Table 1:** Physical quantities and corresponding symbols used in mathematical formulation of conservation equations

**Physical Quantity**	**Symbol**
Fluid mass density	*ρ*
Velocity vector field	**u**
Net mass production rate	
Pressure field	*p*
Viscous stress vector	***τ***
Gravitational acceleration	**g**
Control volume acceleration	**a**
Ventricle wall displacement vector	***δ*_w_**
Differential volume element	*dV*
Differential control surface area element	*dS*
Unit outward surface normal vector	

#### Applying control volume analysis

The computationalist or mathematical modeler will actually attempt to solve these or similar equations. For hydrocephalus, this is extremely difficult and currently requires many assumptions and simplifications, as discussed previously with regard to continuum based models. As experimentalists, we ask, "is it possible to use clinical data to evaluate the balance equations directly?" In this manner, it is possible to gain new information if one can isolate a term which cannot be measured or for which nothing is known. For example, in pipe flow, an applied pressure difference causes flow, so that if the pressure drop is known the flow may be determined. Using control volume anaylsis if the velocity field is known then the pressure drop can be found by applying the momentum equation (2) to the fluid in the pipe. Alternatively, one can use the balance equations to understand what, why, and how changes in the system lead to hydrocephalus. 

**Figure 2:**
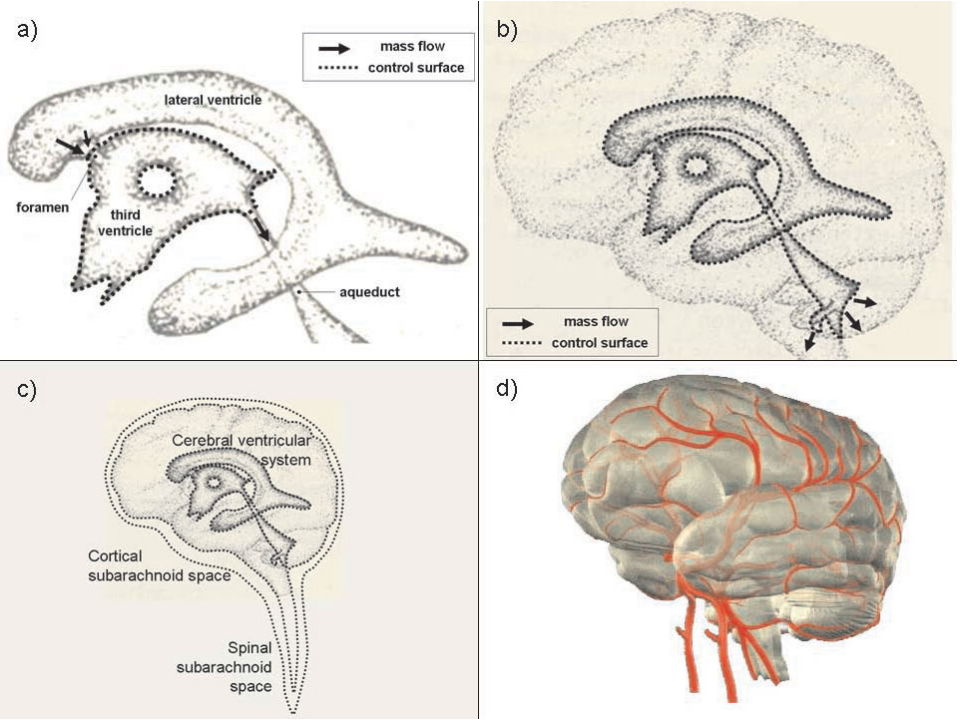
**Examples of in vivo control volumes**. The generality of control volume analysis shown through example of four *in vivo *control volumes enclosing CSF or blood. Control surfaces coincide with the surrounding fluid/tissue interfaces. To completely enclose the specified volumes control surfaces must intersect fluid pathways; it is at these locations where velocity measurements are needed. Individual ventricles, such as the third ventricle a), can be used to determine parameters relevant at the scale of a single ventricle. Similarly, the entire ventricle system b) may be used to estimate CSF production by the choroid plexus. The largest CSF control volume contains all the CSF in a given individual c), in both the ventricular system and the subarachnoid spaces. Blood too must be accounted for, here by a control volume colored red d) which contains all the intracranial arterial, capillary and venous blood. In a) and b) arrows represent the nominal direction of bulk flow and dotted lines in c) represent the control surface.

Control volume analysis is a framework to guide experimental method using the most basic fluid dynamics principles in their simplest form. In addition, using the conservation equations with finite control volumes is an effective way to comprehensively couple spatially separated and physically distinct clinical data sets (e.g. pressure, velocity and volume). It is important to note that as many or as few control volumes may be defined depending on the information desired by the clinician. In general, as a minimum, at least one control volume for the CSF and one for the blood is required to study the important interactions that define the intracranial state, however these spaces may be broken into several smaller control volumes to study the intracranial dynamics on several scales (e.g. see Figure 2). To apply control volume analysis, the first step is to define the control volumes of interest and to write and simplify the conservation equations as they pertain to each individual control volume. With the exact physical meanings of the terms then understood, measurements are taken to obtain these quantities directly.

### Clinical measurements for control volume analysis

Measurements obtained by clinicians have been interpreted through mathematical models and based on relative magnitude and timing of measured flow, pressure and volume waveforms. Other investigations link measurements at single sites in the CSF system to onset or progression of hydrocephalus, such as aqueductal stroke volume [18,29], in an effort to simplify diagnosis and show correlation with measurements and other symptoms. The control volume framework is a general formulation of conservation laws typically used in hydrocephalus modeling, however it represents the most direct and expedient way to interpret clinical data sets.

Velocities of the intracranial contents have been measured throughout the cardiac cycle using velocity encoded cine Phase-Contrast Magnetic Resonance (PC-MR) imaging. *In vivo *blood [17-21,23,25,30,31] and CSF velocities [17-19,21,23-25,29,32-35] have been measured using this technique. PC-MR imaging is commonly used for making velocity measurements like those needed for the control volume formulation. Summing the spatial velocity data, within fluid areas of interest, allows volume and momentum flow rates to be computed directly from the images.

The control volume formulation also requires time resolved pressure measurements at several intracranial sites. CSF pressure measurements have been obtained invasively in dogs [10,36], baboons [25], and humans [37-40]. Invasive ICP monitoring is most commonly achieved with ventricular, subdural, or intraparenchymal microtransducers, however lumbar CSF pressure is commonly measured manometrically in patients with hydrocephalus [37]. Penn *et al*. [36] obtained real time (sampling at 256 Hz) and long term pressure at three intracranial sites: the lateral ventricle, the frontal lobe, and the subarachnoid space. Pressure measurements, such as those made with implanted microtransducers, are needed at the sites where velocity is measured. Blood pressure (and velocity) was measured within the large human arteries using a catheter tip strain gauge transducer (and electromagnetic flowmeter) [41]. Zou *et al*. recently reported blood pressure measurements in the carotid arteries of mongrel dogs using a micro pressure transducer [10]. Blood and CSF pressure measurements will be needed to fully characterize the momentum balance.

**Figure 3:**
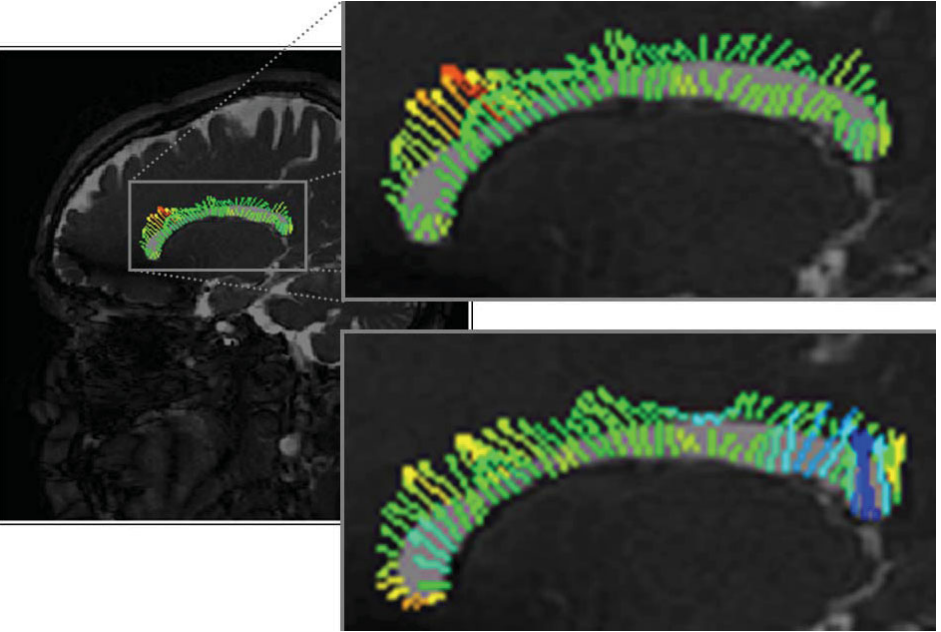
**DPIV volume change estimation technique**. Sagittal MR images at two different times, with Digital Particle Image Velocimetry (DPIV) derived displacement vectors along the lateral ventricle's walls. The vector lengths have been magnified for clarity. Vector color is based on wall normal displacement: red represents distension, blue contraction, and green nominal normal displacement.

In addition to velocity and pressure, the volume (changes) of the fluid spaces also needs to be measured. Several investigators have estimated volume changes directly from medical images [18,19,42,43] or by integrating measured fluid volume flow rate waveforms [20,22,25]. Regardless of the technique used to measure volume change, it is necessary to determine the control volume's extent as a function of time. To supplement previous techniques, we are amending an in-house Digital Particle Image Velocimetry (DPIV) processing program to determine the displacement of the ventricles' walls in MR images. This data can be used, independent of flow measures, to obtain volume changes by adding the change in volume (area change multiplied by slice thickness) for multiple slices. DPIV was developed in the early 1990's to improve upon PIV, which utilized optical interrogation of film negatives to determine velocity magnitude and direction. With DPIV, digital images were post-processed on computers to obtain the same information, automating analysis and making it simpler. This technique was initially developed to accurately and reliably estimate 2D velocity vector fields based on intensity variations within particle images of fluid flow [44]. Our DPIV processing program consists of a two-pass intensity correlation technique to determine, with sub-pixel accuracy, the displacement of features within digital images [45,46]. The resulting regular array of displacement vectors is then interpolated onto the ventricular walls, as shown in Figure 3. Here the calculated wall displacement vectors, ***δ***_w_, (vector lengths magnified for clarity) are colored based on the magnitude of the wall normal displacement, ***δ***_w_· . Red represents a local distension of the ventricular space while blue is a local contraction of the ventricles; nominal motions labeled with green vectors. This data throughout the cardiac cycle allows the volume change to be estimated, and hence for an incompressible fluid (of constant density, *ρ*) the change in mass can be found. The MR imaging was approved by the institutional committee on research with humans and each volunteer gave written consent prior to the imaging session. DPIV represents an additional method to calculate and validate measures of volume change.

Velocity, pressure and volume change represent the types of measurements required, and the methods currently available to obtain the pertinent clinical data sets was discussed. Currently, clinical utilization of these techniques and resulting data sets have been restricted by an inadequate ability to interpret these diverse measurements directly to intracranial volumes of interest.

## Results and Discussion

To illustrate the versatility and generality of the control volume framework consider Figure 2, which shows four control volumes of possible interest to understanding intracranial dynamics. As stated previously several control volumes will be needed to fully quantify the important interactions of the intracranial dynamics. Control volumes of various sizes may be used to study interactions occurring at different scales.

Figure 2a depicts a control volume containing the CSF within the third ventricle. The arrows represent mass flow (in the nominal direction of bulk flow) at the locations where fluid crosses through the control surface, represented by a dotted line. However, it may be more interesting to create a control volume which encloses all the CSF within the ventricular system as shown in Figure 2b, or all the CSF within a given individual as in Figure 2c. Within the latter control volume (Figure 2c) accounting in a single CSF compartment would simplify such that the change in mass is equal to formation minus absorption with no mass flow term. For an incompressible fluid, the control volume equations simplify to the same volume constraint presented in Ref. [3]. Likewise, blood flow and pressure are of interest in hydrocephalus research. A control volume enclosing all (or a portion) of the cerebral blood is shown in Figure 2d and will be important in the overall intracranial accounting. For each of the example control volumes in Figure 2 the same form of the conservation equations are used initially. However, the equations will take different forms depending on the physical meaning of the terms in the equations. It is to say, each control volume will allow different information to be extracted from the conservation equations, making the choice of control volumes to study very important.

The first step in applying control volume analysis, is to define the control volume(s) of interest and to write and simplify the conservation equations as they pertain to each control volume. Then, once the physical meanings of the terms are determined, data is obtained to calculate these quantities directly. Consider applying the proposed framework to the CSF within the third ventricle, as shown in Figure 2a. Control surfaces coincide with the CSF/brain interface (which may move and deform) and 'imaginary' (or mathematical accounting) surfaces across the foramina and aqueduct bounding the third ventricle.

Control volume analysis typically begins with the mass budget (1) because it is simpler to evaluate and leads to constraints which carry over to the momentum equation. Term (*i*) in Equation (1), in this case, represents the change of CSF (mass) in the third ventricle. Volume change may be estimated using a variety to techniques, as mentioned previously. Generally, volume (change) measurements are based on processing of image data, be it velocity or static images. As mentioned previously, an in-house DPIV processing program is being used to calculate the displacement of the ventricular walls, as shown in Figure 3, directly from MR images. Reliability of volume estimates depend on image resolution, the volume change amplitude and technique limitations.

PC-MR produces images in which the pixel intensity is proportional to velocity. Typically, for ease of computation, through plane velocities are obtained when volume flow information is desired. Velocity (image) data taken throughout the cardiac cycle allows volume flow waveforms to be computed, as has been done previously by numerous investigators for various fluid pathways [17-20,22,25,29,31]. In this example, for the third ventricle, CSF flows through the control surface at the interventricular foramina (of Monroe) from the paired lateral ventricles and at the aqueduct/ventricle 'boundary'. PC-MR measurements are possible at these locations (e.g. see [17,21]) and allow direct measurement of the mass flows crossing the control surface, the physical representation of (*ii*) in the mass budget. It can be seen then that (*iii*), the net production/absorption rate in the third ventricle, could be estimated with reliable measurements of the other two terms, namely the time varying CSF mass (or volume) of the third ventricle and the net CSF mass (volume) flow through the control surfaces, respectively.

We next turn to the momentum equation (2) which represents a balance of forces on the control volume. The major difference between mass and momentum conservation is the inclusion of pressure terms in the momentum budget, as stated previously. The time-rate of change of momentum and momentum flow (or force due to fluid flow), (*i*) and (*ii*) in (2), can be found in a similar fashion to the corresponding terms in the mass budget. Minimally pressure measurements are needed at the same locations on the control surface as the flow measurements, namely where the foramina and aqueduct meet the third ventricle. Due to inadequate resolution of pressure sensors compared to the small pressure differentials between communicating fluid cavities, pressure differences are not likely to be measured between two points on the control surface. However, if the other terms are reliably measured, and pressure at one point on the control surface is measured, the pressure at another location can be determined from the momentum balance. The remaining pressure and viscous forces on the ventricle's walls (which are difficult to measure and generally small e.g. [25]) may be grouped into a 'resistive' term. The body forces (i.e. gravity and acceleration) in equation (3) can be found as long as the volume (change) of the system is known, since these terms are constant throughout the control volume for any head motion. Integral control volume analysis, then, naturally incorporates the changes which take place during dynamic postural changes, which can have a significant impact on normal intracranial dynamics as recently shown [47]. So with the flow and pressure measurements, as well as volume (change) estimates, the 'resistance' in the system can be isolated for examination. If the 'resistance' is assumed or found to be small, then pressure at a location where measurements may not be possible can be found using the momentum balance equation. Momentum conservation, as presented in control volume analysis, represents a new way to account for pressure and relate it to flow.

One can see, then, that the power of control volume analysis lies in the fact that only terms accounting for net changes within the system and flow and pressure at the control surface are encountered. Thus the use of control volumes preclude the need for velocity and pressure field information everywhere in the cranium, greatly simplifying the analysis. Measurements are directly tied to the physical meaning of terms in the conservation equations, (1) and (2), and obtained using a blend of modern techniques such as MR imaging, implantable micro-pressure-transducers, and DPIV processing. With this approach, measurements taken at different locations can be related through the control volume budgets directly using fundamental conservation principles. In addition, interactions between systems and subsystems can be studied, by using several control volumes of various scales, and are potential locations for key deviations from equilibrium.

## Conclusion

Current understanding and accounting of intracranial dynamics and progression to disorder is limited by the availability of clinical data and the ability of the investigator to interpret the data in a relevant, physically meaningful way. The control volume formulation incorporates diverse measurements into a fundamental fluid dynamic conservation analysis, yielding a direct mechanical interpretation of the data. The application of control volume analysis to intracranial control volumes was discussed and suggestions are made for utilizing this technique in future studies of intracranial dynamics.

## Competing interests

The authors declare that they have no competing interests.

## Authors' contributions

BC and TW contributed equally to this work by formulating the control volume analysis to general intracranial control volumes. BC drafted the manuscript. TW proposed the initial idea of using control volume analysis to guide and interpret intracranial data. SV and AV worked on the DPIV ventricle dilatation processing technique. All authors have read and approved the final version of the manuscript.
